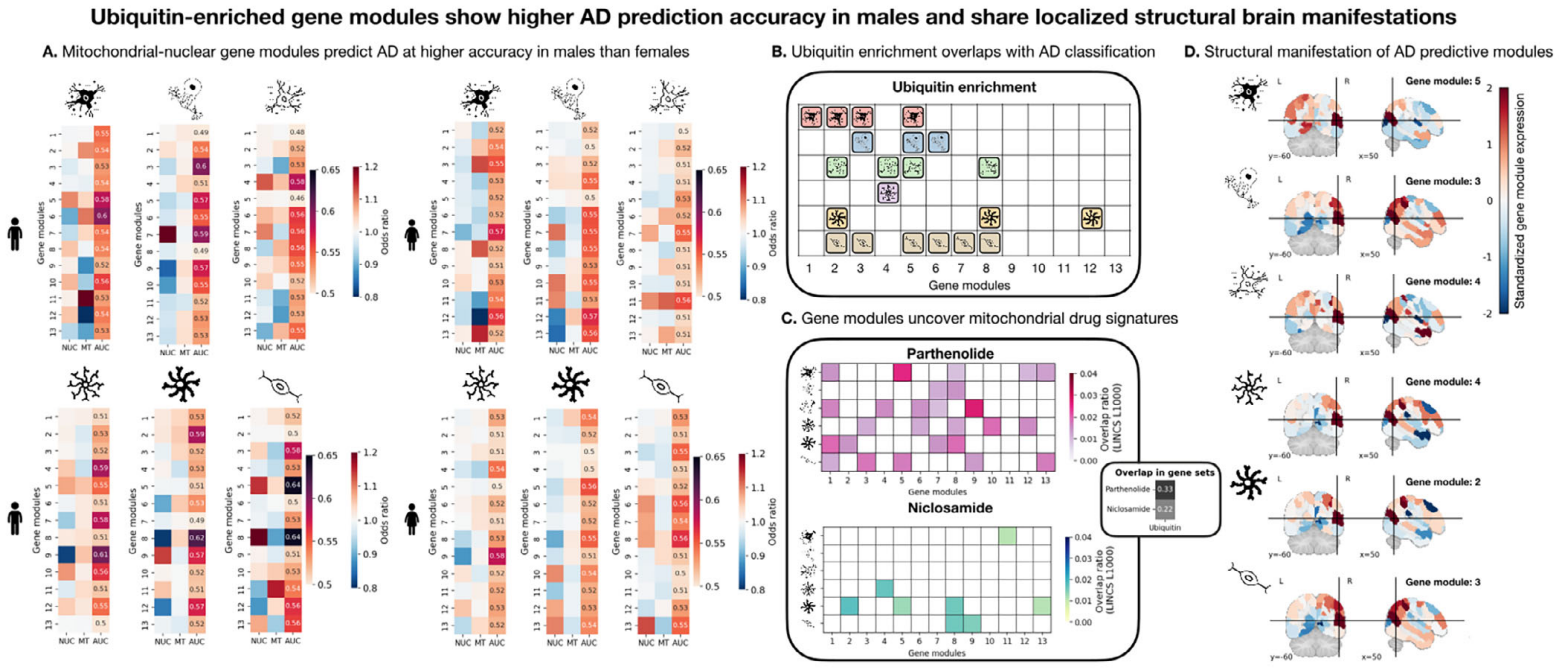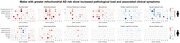# Mitochondria‐nucleus crosstalk characterizes Alzheimer's disease across 1,5 million brain cells

**DOI:** 10.1002/alz70855_096286

**Published:** 2025-12-23

**Authors:** Chloé Savignac, Liam Hodgson, Jack Stanley, Anwesha Bhattacharya, Danilo Bzdok

**Affiliations:** ^1^ Mila ‐ Quebec Aritificial Intelligence Institute, Montreal, QC, Canada; ^2^ McGill University, Montreal, QC, Canada

## Abstract

**Background:**

Emerging insight from stem cell research reinforces Alzheimer's disease (AD) to affect mitochondrial protein expression. Compelling new evidence points to mitochondrial reactive oxygen species (ROS) as potential driving player in Aβ toxicity, mediated through glial cells and ultimately impacting neuronal health. A comprehensive understanding of how oxidative phosphorylation variations relate to cell function remains largely unexplored, especially through a cell type lens.

**Method:**

Leveraging today's largest single‐nucleus RNA sequencing dataset of AD, we unveil how cell‐type‐specific mitochondrial alterations reverberate in the nuclear transcriptome, in 424 AD patients and healthy controls from ROSMAP. By adopting a supervised latent factor modelling approach, we identified distinct gene modules capturing unique aspects of the mitochondrial crosstalk in 6 major brain cell types across 5,427 nuclear and 13 mitochondrial genes.

**Result:**

We found that nuclear‐mitochondrial crosstalk varies distinctly with cell identity, reflecting metabolic demands and functional specialization. In neurons and oligodendrocytes, ATP synthase (complex V) takes a central role, whereas type 1 NADH dehydrogenase (complex I) is more prominent in astrocytes, microglia, and OPCs. Screening across >1 million gene expression profiles from ∼20,000 drug perturbations identified mitochondrial‐nuclear signatures that resemble those activated by parthenolide and niclosamide—two chemical compounds previously associated with oxidative stress and cytotoxicity via ubiquitination—as most predictive of AD. Microglia and OPCs achieved the highest overall classification accuracy, with stronger predictive performance observed in males than in females. Mapping gene module expressions to the Allen Human Brain Atlas revealed shared whole‐brain patterns highlighting the precuneus, which we implicated in ubiquitin‐cascade‐enriched modules. Clinical phenotyping revealed that males with higher AD risk, as indicated by their mitochondrial‐nuclear scores on glial gene modules, exhibited a greater pathological burden, including higher amyloid load, Parkinson's‐like symptoms, and neuroticism‐related traits. Finally, by comparing our findings with 2.5 million CRISPRi‐based perturbations, we identified neural signatures associated with female‐biased transcription factors and fatty acid biosynthesis, while glial signatures were linked to DNA damage and oxidative stress.

**Conclusion:**

By integrating multiple layers of biological data from established reference atlases, our analysis of mitochondria‐nuclear crosstalk revealed distinct transcriptional signatures associated with AD risk in glial and neural cells, with these associations exhibiting sex‐biased patterns.